# High-Throughput Genotyping in Metastatic Esophageal Squamous Cell Carcinoma Identifies Phosphoinositide-3-Kinase and BRAF Mutations

**DOI:** 10.1371/journal.pone.0041655

**Published:** 2012-08-03

**Authors:** Chi Hoon Maeng, Jeeyun Lee, Paul van Hummelen, Se Hoon Park, Emanuele Palescandolo, Jiryeon Jang, Ha Young Park, So Young Kang, Laura MacConaill, Kyoung-Mee Kim, Young-Mog Shim

**Affiliations:** 1 Division of Hematology-Oncology, Department of Medicine, Samsung Medical Center, Sungkyunkwan University School of Medicine, Seoul, Korea; 2 Center for Cancer Genome Discovery, Dana-Farber Cancer Institute and Harvard Medical School, Boston, Massachusetts, United States of America; 3 Department of Pathology, Samsung Medical Center, Sungkyunkwan University School of Medicine, Seoul, Korea; 4 Department of Thoracic Surgery, Samsung Medical Center, Sungkyunkwan University School of Medicine, Seoul, Korea; University of Navarra, Spain

## Abstract

**Background:**

Given the high incidence of metastatic esophageal squamous cell carcinoma, especially in Asia, we screened for the presence of somatic mutations using OncoMap platform with the aim of defining subsets of patients who may be potential candidate for targeted therapy.

**Methods and Materials:**

We analyzed 87 tissue specimens obtained from 80 patients who were pathologically confirmed with esophageal squamous cell carcinoma and received 5-fluoropyrimidine/platinum-based chemotherapy. OncoMap 4.0, a mass-spectrometry based assay, was used to interrogate 471 oncogenic mutations in 41 commonly mutated genes. Tumor specimens were prepared from primary cancer sites in 70 patients and from metastatic sites in 17 patients. In order to test the concordance between primary and metastatic sites from the patient for mutations, we analyzed 7 paired (primary-metastatic) specimens. All specimens were formalin-fixed paraffin embedded tissues and tumor content was >70%.

**Results:**

In total, we have detected 20 hotspot mutations out of 80 patients screened. The most frequent mutation was PIK3CA mutation (four E545K, five H1047R and one H1047L) (N = 10, 11.5%) followed by MLH1 V384D (N = 7, 8.0%), TP53 (R306, R175H and R273C) (N = 3, 3.5%), BRAF V600E (N = 1, 1.2%), CTNNB1 D32N (N = 1, 1.2%), and EGFR P733L (N = 1, 1.2%). Distributions of somatic mutations were not different according to anatomic sites of esophageal cancer (cervical/upper, mid, lower). In addition, there was no difference in frequency of mutations between primary-metastasis paired samples.

**Conclusions:**

Our study led to the detection of potentially druggable mutations in esophageal SCC which may guide novel therapies in small subsets of esophageal cancer patients.

## Introduction

Esophageal cancer is the sixth most common cause of cancer worldwide and the incidence rates vary according to sex, countries, and histological types [1]. While esophageal adenocarcinoma is more frequent in Western countries, squamous cell carcinoma is the dominant histologic subtypes globally [1], [2]. Esophageal cancer is a highly aggressive disease and the 5-year survival rate is approximately 15% [3]. Approximately 50% of patients show distant metastasis and half of the remaining patients who initially present with locoregional disease eventually develop distant metastases. In case of metastatic disease, median survival is less than a year despite of palliative chemotherapy [3].

The role of chemotherapy in metastatic esophageal cancer has not yet been documented through phase III trials and median survival is less than 10 months despite conventional chemotherapy [4], [5]. Given the high incidence of metastatic esophageal squamous cell carcinoma (SCC) in East Asia and the poor prognosis with ineffectiveness of conventional chemotherapy, there is an urgent need to find novel targeted agents to improve treatment outcomes. Nowadays, there are somatic mutations known to be predictive of drug sensitivity or drug resistance. We have adapted a high-throughput genotyping platform to determine the mutation status of a large panel of known cancer oncogenes to identify the subsets of esophageal cancer patients who may potentially benefit from targeted therapy [6], [7]. The genotyping platform, termed OncoMap, employs mass spectrometric-based genotyping technology (Sequenom) to identify 471 oncogenic mutations in 41 commonly mutated genes (Table S1). In this study, we screened for somatic mutations using high-throughput technology in an attempt to identify potential target populations for molecularly targeted agents in esophageal SCC.

## Results

**Figure 1:**
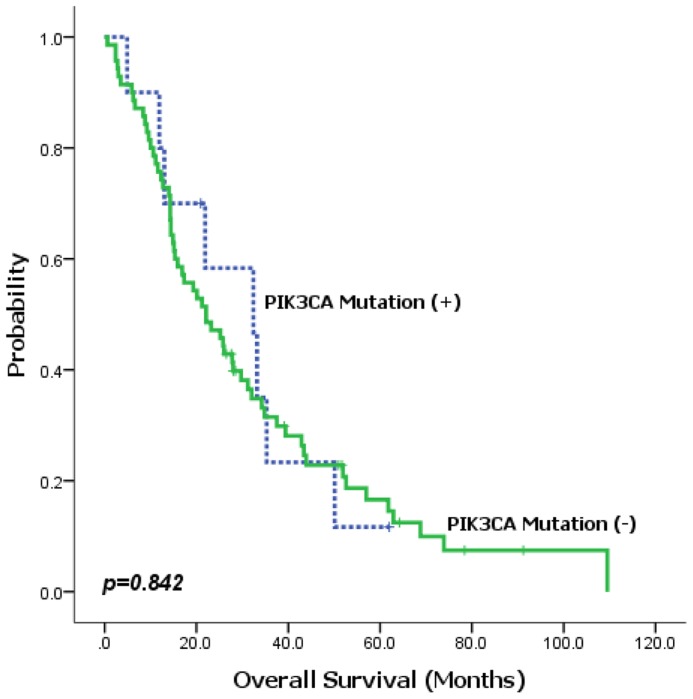
Overall survival according to PIK3CA mutation status.

In this study, we examined 87 esophageal squamous cell carcinoma samples from which 70 were from primary tumor sites and 17 from metastatic sites (80 patients in total). We included 7 pairs of primary-metastatic esophageal SCC. All patients had histologically confirmed squamous cell carcinoma and all patients received fluoropyrimidine/platinum chemotherapy between January 2008 and January 2010. The baseline characteristics are summarized in Table 1. Among the 87 FFPE samples tested, 23 mutations were identified and validated. The 11 mutations that were identified involved 6 genes (Table 2). Of 11 mutations, 4 mutations were located in PIK3CA, thus the most frequently observed somatic mutations in esophageal SCC. All 4 hotspots in PIK3CA, E545K, E542K, H1047R, H1047L, are known to be oncogenic in various tumor types [8]–[11]. There was no significant association between anatomic location and PIK3CA mutations. Of the 9 cases with PIK3CA mutations, 4 (44.4%) patients had mid-esophagus, one (11.1%) had upper-esophagus and 4 (44.4%) had distal esophageal cancer. Next, we examined the impact of PIK3CA mutations on treatment outcome after fluoropyrimidine/cisplatin chemotherapy. Overall, there was no significant difference in overall survival between the two groups (PIK3CA mutation (+) vs (−), 8.0 and 4.4 months, respectively, *P* = 0.842) (Figure 1). TP53 mutations were observed in 3 cases (3.5%). In addition, we found one BRAF V600E (1 of 80 patients, 1.2%) and one CTNNB1 D32N (1 of 80 patients, 1.2%) and one EGFR P733L (1 of 80 patients, 1.2%).

**Table 1:** Baseline characteristics of patients.

			N = 80	% of Total Case
Sex		Male	75	93.7
		Female	5	6.3
Age (year)			43–74 (Median: 63)	
Tissue Specimen		Primary	70	80.4
		Metastatic	17	19.6
		Both	7	8.0
Metastatic Site		Lymph node	9	50.6
		Lung	2	11.7
		Liver	2	11.7
		Larynx	1	6.5
		Small intestine	1	6.5
		T/L spine	1	6.5
		Femur	1	6.5
Differentiation		Well	3	3.4
		Moderate	38	43.7
		Poor	8	9.2
		Others	38	43.7
Anatomic sites		Upper third	7	8.7
		Middle third	37	46.3
		Lower third	26	32.5
		Upper to middle	2	2.5
		Mid to lower	8	10
Primary resection		Done	51	63.7
		Not done	29	36.3
Chemotherapy		Palliative (fluoropyrimidine/platinum-based chemotherapy)	78	
		Others	2	

**Table 2:** Frequency of somatic mutations in esophageal squamous cell carcinoma.

Gene	Amino acid	Primary site		Metastatic site	%††
		(n = 70)		(N = 17)†	
**PIK3CA**	E545K	2	0		11.5
	E542K	2	0		
	H1047R	4	1		
	H1047L	1	0		
**MLH1**	V384D	5	2		8.0
**TP53**	R306	1	0		3.5
	R175H	1	0		
	R273C	1	0		
**BRAF**	V600E	1	0		1.2
**CTNNB1**	D32N	1	0		1.2
**EGFR**	P733L	1	0		1.2
**TOTAL**		20	3		26.6

† 7 cases were pairs of primary and metastatic organ.

†† Percentage of total number of patients (N = 80).

We included 7 pairs of primary-metastatic tumor specimens in order to evaluate the concordance rate of somatic mutations between primary and metastatic tumors. We observed 100% concordance rate of mutations between primary-metastatic pairs (Table 3). There was one case (MLH1 V384D) that harbored mutation in both of primary and metastatic tumor tissues. The remainders showed no mutations from primary or metastatic sites.

**Table 3:** Concordance between primary and metastatic paired samples.

Patient No	Primary	Metastatic
9	Negative	Negative
22	Negative	Negative
29	Negative	Negative
31	Negative	Negative
40	Positive :MLH1_V384D	Positive :MLH1_V384D
69	Negative	Negative
77	Negative	Negative

## Discussion

In this study, we performed high-throughput screening in metastatic, unresectable esophageal SCC with the aim of identifying mutations for molecularly targeted agents. There are very limited options to treat metastatic esophageal SCC and clinical trials incorporating novel agents are severely limited. Overall, 20 (25%) of 80 patients screened using OncoMap had somatic mutations. We identified that 11% of metastatic esophageal SCC had PIK3CA mutations in exon 9 (E542K, E545K) and exon 20 (H1047R, L) and that 1% of the patients harbored BRAF V600E mutations.

There are scarce reports on PIK3CA mutations in esophageal SCC. There is one study which directly sequenced tumor DNA isolated from FFPE and identified 4 of 35 (11.8%) esophageal SCC to have PIK3CA mutation [12]. Based on these results, identification of PIK3CA mutation may define a subset of patients who may be potential candidates for PIK3CA inhibitor treatment [13]. In addition, mTOR inhibitors and AKT inhibitors are currently under the investigations to target subsets of tumors with aberrant PIK3 pathway [14], [15]. A recent preclinical study had demonstrated that LY294002, a PI3K inhibitor, reduced the proliferation of the esophageal cancer cell line *in vitro* [16].

There was one patient (1 of 80) who harbored an oncogenic BRAFV600E mutation in our series. To the best of our knowledge, this is the first report on the BRAF mutation in esophageal SCC. BRAF has taken center stage due to the discovery that it is mutated in prevailing human cancers, including 60% of malignant melanomas and 5%–15% of colon, ovarian, and thyroid carcinomas [17]. The most common mutation is V600E, which dramatically enhances BRAF enzyme activity, and thus induces tumorous transformation. Recently, vemurafenib, a potent inhibitor of mutated BRAF [18], has demonstrated a survival benefit in metastatic melanoma with BRAFV600E mutation [19]. Although preclinical study on efficacy of BRAF inhibitors is yet to be tested in esophageal SCC, prospective screening for the presence of BRAF mutation should be actively considered in esophageal SCC given the lack of treatment options for these patients.

TP53 is a tumor suppressor gene that encodes protein mediating cell apoptosis. Loss-of-function mutation of TP53 is one of the most common features of human cancers [20]. In COSMIC database published by Sanger institute, TP53 mutation is the most common one among esophageal SCC (50%). Other investigators have reported the frequency of TP53 mutation in the range from 35% to 80% [20], [21]. However, the frequency of TP53 mutation was lower (3.4%) in our series when compared to those reported in previous studies. The lower rate of TP53 mutations in this study is likely due to the fact that TP53 was genotyped at only a 7 loci, rather than sequenced, and thus OncoMap is likely to miss many such events. This is the first report of assessing esophageal cancers from an Asian population and therefore there is no independent validation series available. The proportion of analyzed specimens from lower thirds portion of esophagus in our report is 32.5%. As revious report, the incidence of esophageal squamous cell carcinoma is thought to be approximately 50% [3], the relatively fewer portion of specimen numbers could lead the bias lowering mutation detection rates those are frequently found in lower thirds esophagus. As mentioned above, the rate of TP53 mutations were reported exceptionally lower that previous data such as COSMIC database. While the only detected muation in lower third portion of esophagus is TP53 in the results from COSMIC Database, we reported three detected TP 53 mutations located in upper and middle thirds, suggesting omission of TP53 mutation detection possilby located in distal esophagus.

OncoMap platform, which we used for mutation screening, is a reasonable substitute for direct sequencing method, not only in fresh/frozen tissues but informalin-fixed paraffin-embedded specimen (FFPE) [7]. The sensitivity and specificity of OncoMap is approximately 89.3% and 99.4%, respectively when FFPE tissue is used and those are comparable with the result from OncoMap analysis using fresh frozen tissue [7]. One of the most remarkable advantages of OncoMap is that it enables us to screen hundreds of hotspot mutations using FFPE at reasonable cost [7]. In addition, whole genome sequencing using fresh frozen tissues may not always be feasible in metastatic solid tumor patients considering poor medical condition and accessibility of tumor biopsy. Hence, until whole genome sequencing or whole exome sequencing is robustly confirmed in FFPE, clinicians have to utilize a reliable way to screen for druggable hotspot mutations. OncoMap has been shown to be a reliable method using tumor DNA extracted from FFPE to screen for somatic mutations in multiple solid tumor types [7], [22], [23].

The paradigm of cancer treatment is rapidly changing from disease-specific type to target-specific approach such as testing the efficacy of BRAF inhibitors in BRAF mutant solid tumors regardless of primary sites. Using FFPEs, we successfully screened 80 metastatic esophageal SCC and have identified PIK3CA mutation (11.5%) and BRAF mutation (1.2%) as potential targets for further therapeutic development. Although the PIK3CA mutation itself did not affect patients’ survival, effective targeting the mutations can be associated with survival benefit. Based on this study, we plan to propose clinical trials with PI3K inhibitors and/or BRAF inhibitors.

## Methods

### Patients

We analyzed total 87 tissue specimens obtained from 80 patients who had pathologically confirmed squamous SCC and treated in Samsung Medical Center, Korea from January 2008 to December 2010. All patients had recurrent or metastatic disease and had received 5-fluoropyrimidine/platinum-based chemotherapy. Of the 87 tumor tissue specimens analyzed, 70 specimens were from primary sites and 17 specimens were from metastatic sites. All primary tumor and metastatic tumor samples were obtained from formalin-fixed paraffin-embedded tumor specimens based on 80% cutoff for tumor sample purity from a single institute. The quality of all DNA samples was ensured by independent quantification and quantitative PCR. The study was conducted after the approval from the Samsung Medical Center Institutional Review Board (SMC IRB). The primary tumor samples were all collected from Samsung Medical Center. The study was approved by the SMC IRB for informed consent waiver using archival tissues with retrospective clinical data and all data was de-identified.

### Selections of Oncogene Mutations and Genotyping

Our current OncoMap v4 interrogates 471 mutations in 41 genes that are relevant for cancer (Table S1). We selected various known oncogenes and tumor suppressor genes based on previous published literatures [6], [7]. OncoMap v4 what we used for genotyping analysis here is an expansion of Oncomap v1 previously mentioned by Macconaill et al [7]. We selected the candidate somatic mutations in OncoMap v4 based on literature reviews and frequently detected mutations reported in the COSMIC Database. Genomic DNA was quantified using Quant-iT™ PicoGreen® dsDNAAssay Kit (Invitrogen) per manufacturer’s protocol. 250 ng DNA was used for a mutation analysis using Oncomap mass spectrometric genotyping based on the Sequenom MassARRAY® technology and (Sequenom Inc, San Diego, CA) performed as previously described high-throughput oncogene mutation profiling in human cancer with some modifications [6], [7]. 100 ng of tumor-derived genomic DNA was subjected to whole genome amplification (WGA). Next, up to 18-multiplexed PCR was performed on tumor genomic DNA to amplify regions harboring loci of interest. After denaturation, PCR products were incubated with the probes that anneal immediately adjacent to the query nucleotide and mass spectrometric genotyping using iPLEX chemistries was performed (Sequenom Inc, San Diego, CA) extending the probes with 1 base in the presence of chain-terminating di-deoxynucleotides that generate allele-specific DNA products. The extension products were spotted onto a specially designed chip and analyzed by MALDI-TOF mass spectrometry to determine the mutation status based on the difference in mass of the mutant and wild type base.

Next, an automated mutation calling algorithm was performed to identify candidate mutations. Putative mutations were further filtered by a manual review and selected for validation using multi-base homogenous Mass-Extend (hME) chemistry with a maximum pooling of 6 assays on the remaining 150 ng DNA of each sample. Primers and probes used for hME validation were designed using the Sequenom MassARRAY® Assay Design 4.0 software, applying default multi-base extension parameters.

Only mutations found in iPLEX and confirmed by hME were considered as ‘validated mutations’. iPLEX candidate mutations that were not confirmed by hME were considered as invalidated and were not reported. Examples of all detected mutations were confirmed by standard, bidirectional Sanger sequencing.

## Supporting Information

Table S1 List of Genes screened for in OncoMap 4.0.
(DOCX)
